# Establishing the “Fit” between the Patient and the Therapy: The Role of Patient Gender in Selecting Psychological Therapy for Distressing Voices

**DOI:** 10.3389/fpsyg.2016.00424

**Published:** 2016-03-31

**Authors:** Mark Hayward, Luke Slater, Katherine Berry, Salvador Perona-Garcelán

**Affiliations:** ^1^School of Psychology, University of SussexBrighton, UK; ^2^Sussex Education Centre, Sussex Partnership NHS Foundation TrustHove, UK; ^3^School of Psychological Sciences, University of ManchesterManchester, UK; ^4^Personality, Evaluation and Psychological Treatment Department, University of SevilleSeville, Spain; ^5^Virgen del Rocío Outpatient Mental Hospital, University Hospital Virgen del RocíoSeville, Spain

**Keywords:** auditory hallucinations, distressing voices, psychological therapy, mindfulness, gender, emotional reaction, behavioral response

## Abstract

The experience of hearing distressing voices has recently attracted much attention in the literature on psychological therapies. A new “wave” of therapies is considering voice hearing experiences within a relational framework. However, such therapies may have limited impact if they do not precisely target key psychological variables within the voice hearing experience and/or ensure there is a “fit” between the profile of the hearer and the therapy (the so-called “What works for whom” debate). Gender is one aspect of both the voice and the hearer (and the interaction between the two) that may be influential when selecting an appropriate therapy, and is an issue that has thus far received little attention within the literature. The existing literature suggests that some differences in voice hearing experience are evident between the genders. Furthermore, studies exploring interpersonal relating in men and women more generally suggest differences within intimate relationships in terms of distancing and emotionality. The current study utilized data from four published studies to explore the extent to which these gender differences in social relating may extend to relating within the voice hearing experience. The findings suggest a role for gender as a variable that can be considered when identifying an appropriate psychological therapy for a given hearer.

## Introduction

The experience of hearing distressing voices has recently attracted much attention in the literature on psychological therapies. This interest seems partly motivated by a desire to improve upon the moderate effects of Cognitive Behavior Therapy for Psychosis (CBTp; Jauhar et al., 2014; van der Gaag et al., 2014). The cognitive-behavioral model of voice hearing proposes that beliefs held about voices influence levels of distress and how people respond to them (e.g., Chadwick and Birchwood, 1994). Hence, CBTp for voices aims to work with these beliefs, in order to reduce distress and disturbance. However, there has recently been a shift from conceptualizing a voice as a sensory stimulus that the hearer holds *beliefs about*, to a voice as a person-like stimulus which the hearer has a *relationship with* (Hayward et al., 2011). This conceptualization is consistent with personal accounts of hearing voices where the hearers typically personify voices and report having relationships with them as if they were real people (Escher and Romme, 2012; McCarthy-Jones et al., 2014a). Understanding voices within relational frameworks has resulted in a new “wave” of therapies for voices that focus upon the “experience” of relating to and with distressing voices. Many of these developments draw on the principles of CBTp, but also reflect additional influences.

Max Birchwood and colleagues have developed a treatment based on a relational approach (Social Rank Theory) to specifically target command hallucinations. Cognitive Therapy for Command Hallucinations (CTCH) is based on the finding that perceptions of voice power and superiority reflect appraisals of broader social status (Birchwood et al., 2004) and aims to reduce voice-related distress through altering the power balance between voice hearer and voice by increasing the power of the hearer. A recent large multicenter trial of CTCH reported reduced compliance and perceived voice power compared with treatment as usual (Birchwood et al., 2014). However, it remains to be determined whether these positive findings generalize to other types of voices and beliefs about voices.

Hayward and colleagues have extended the relational focus beyond power to include issues of proximity and intimacy. Drawing upon Birtchnell's (1996, 2002) Relating Theory, they have demonstrated similarities between voice and social relationships on the axes of both power and proximity (Hayward, 2003). Additionally, distant relating to the voice has consistently been associated with distress, suggesting that a safe distance from a voice cannot be achieved (Hayward et al., 2008; Sorrell et al., 2010). Relating Therapy (RT) has been developed to assist hearers to engage assertively with both their voices and other people within their social worlds. Whilst the effectiveness of RT needs to be addressed within randomized controlled trials, findings to date from case series analyses and qualitative research are encouraging and suggest that issues of *both* power *and* proximity can influence distress and may be amenable to therapeutic modification (Hayward et al., 2009; Hayward and Fuller, 2010).

A further way to change voice-hearers' maladaptive relationships with their voices may be to disengage from responding to voices interpersonally through either Mindfulness-based (e.g., Dannahy et al., 2011) or Acceptance and Commitment Therapy (ACT; Thomas et al., 2013) approaches (see Strauss et al., 2015, for a review of mindfulness-based interventions for voices). Evidence from small uncontrolled studies with voice-hearers show that mindfulness based approaches are associated with significant improvements in psychological well-being (Chadwick et al., 2009; Dannahy et al., 2011). ACT is a similar approach to mindfulness which has been applied to help people disengage from responding to voices automatically and to regard them simply as patterns of words (Thomas et al., 2013). Findings from randomized controlled trials of ACT for psychosis are thus far promising (Bach and Hayes, 2002; Gaudiano and Herbert, 2006) and further work is underway to develop the evidence base (Thomas et al., 2014).

Although the evidence base for relationally based therapies is thus far encouraging, such therapies may have limited impact if they do not ensure a “fit” between the profile of the hearer and the therapy (the so-called “What works for whom” debate). It is possible that theoretical models and therapeutic approaches informed by interpersonal perspectives may be relevant to only a sub-set of hearers, and indeed, that different interpersonal approaches may be more or less suited to different types of voice-hearers or voice-hearing experiences (Hayward et al., 2011).

CBT based approaches have evolved to be focused on individualized case conceptualization as the key means of determining therapeutic direction (Tarrier and Johnson, 2006). However, this process would benefit significantly from research-based principles to aid in conceptualizing individual differences related to voice experience and prioritizing therapy targets (van der Gaag et al., 2014). Gender is one aspect of both the voice and the hearer (and the interaction between the two) that may be influential when selecting an appropriate therapy. Consequently, it is surprising that so little attention has been paid to gender within the literature, especially the gender of the hearer. The existing literature on hearer gender suggests that differences are evident: voice hearing is more common in women (Rector and Seeman, 1992; Murphy et al., 2010), who have a higher frequency of voices (Sharma et al., 1999) and a more delusional interpretation of these experiences (Gonzalez et al., 2008); whereas voice hearing begins at an earlier age for men and is more persistent (Gonzalez et al., 2008). With respect to gender of the voice, the “dominance” of the male voice seems evident for both men and women (Legg and Gilbert, 2006). No differences have been reported across genders with respect to beliefs about voices (e.g., Thomas et al., 2015).

Accepting the similarities between relating to voices and social others described earlier, the fit between hearer and relationally based therapy is likely to be influenced by gender differences in interpersonal relating more generally. In terms of proximity of relating, there is evidence of gender differences in self-reported desire for closeness in intimate relationships, with men seeking greater distance and women greater closeness (Feeney, 1999; Birtchnell and Evans, 2004). There is also evidence of gender differences in emotional reactivity (increased in women, Skowron and Dendy, 2004), and in intensity (with women experiencing emotions more intensely than men, Searle and Meara, 1999). There is also good evidence from large cross-cultural reviews and meta-analyses that men report higher attachment avoidance than women whereas women report higher attachment anxiety than men in adult attachment relationships, providing further support for gender differences in interpersonal relating (Schmitt, 2003; Del Giudice, 2011).

In summary, the gender differences in relating outlined above suggest that females might be more inclined to engage with voices and exhibit strong emotional reactions when relating to them. By comparison, male hearers might seek to distance themselves from voices in order to limit exposure to associated emotions. We aimed to test hypothesized relationships between gender and ways of relating and responding to voices in a sample of voice hearers drawn from four independent studies, and tested three specific hypotheses:

*Hypothesis 1—Female hearers will engage more with their predominant voice, when compared to male hearers—evidenced by significantly higher scores on the engagement scales of the Beliefs About Voices Questionnaire (BAVQ-R)*.

*Hypothesis 2—Male hearers will respond to their predominant voice with greater resistance, when compared to female hearers—evidenced by significantly higher scores on the resistant scales of the Beliefs About Voices Questionnaire (BAVQ-R)*.

*Hypothesis 3*—*Male hearers will relate to their dominant voice from a position of greater distance, when compared to female hearers—evidenced by significantly higher scores on the distance scale of the Voice and You (VAY)*.

## Methods

### Participants

Data were drawn from the baseline data of four published studies: Dannahy et al. (2011), Hayward (2003), Hayward et al. (2008), and Sorrell et al. (2010). In total, data from 148 participants was included in this study, 75 of whom were women. Participants had a mean age of 39.56 years (*sd* = 9.76), had been hearing voices for ~14.84 years (*sd* = 10.43), and were all prescribed antipsychotic medication. Diagnoses were obtained from either participants or case notes and were as follows: 117 schizophrenia, 8 schizoaffective disorder, 5 psychosis, 8 psychotic depression, 1 bipolar disorder, 4 personality disorder, 1 PTSD, and 4 were unknown.

Most participants contributed data concerning both emotional/behavioral responses to their voices, and relating to voices. Additionally, some participants (from Dannahy et al., 2011) contributed only data about their relational responding to voices, and some participants contributed only data on emotional/behavioral responses to voices (from Hayward, 2003). See Table 1 for the profile of participants and their contributions to this study.

**Table 1:** Participant demographics and contributions to BAVQ-R^*^ and VAY analyses.

	**Hayward, 2003**		**Hayward et al., 2008**		**Sorrell et al., 2010**		**Dannahy et al., 2011**		**Combined**	
Male (*N*)	16		16		19		22		73	
Female (*N*)	11		11		13		40		75	
Age – *M*(SD)		39.52 (10.73)		39.52 (10.54)		38.1 (9.3)		41.1 (9.2)		39.56 (9.76)
Diagnosis										
Schizophrenia	26		16		20		55		117	
Schizoaffective disorder	0		4		4		0		8	
Psychosis	0		0		0		5		5	
Psychotic depression	1		3		4		0		8	
Bipolar disorder	0		0		1		0		1	
Personality disorder	0		3		0		1		4	
PTSD	0		0		0		1		1	
Unknown	0		1		3		0		4	
Duration of voice hearing (years)		12.59 (8.30)		15.40 (13.27)		17.06 (10.70)		14.3 (9.7)		14.84 (10.43)
Participants contributing to total VAY	0		26		31		62		119	
Participants contributing to total BAVQ-R	27		27		32		30		116	

* *BAVQ-R data taken from Dannahy et al. (2011) did not appear in the original publication*.

Ethics approval for the four published studies was granted by the National Research Ethics Service within the United Kingdom. Research Governance approval was granted by the appropriate Mental Health Trust within the National Health Service.

### Measures

#### The beliefs about Voices Questionnaire–revised (BAVQ-R)

Emotional and behavioral responses to voices were measured using the BAVQ-R. The BAVQ-R is a 35-item questionnaire comprising five subscales: three assessing beliefs about the dominant voice in the form of malevolence (6 Items), benevolence (6 Items) and omnipotence (6 Items); and two assessing resistance (9 Items) and engagement (8 Items). The resistance and engagement subscales are each further divided into *emotional* and *behavioral* responses to the voice. All items are measured on a four-point scale (0–3). Internal reliability for the BAVQ-R has been shown to be good, with Cronbach's α scores for each of the primary subscales as follows: 0.84 malevolence, 0.88 benevolence, 0.74 omnipotence, 0.85 resistance, and 0.87 for engagement (Chadwick et al., 2000).

#### Voice and You (VAY)

The VAY is a 28-item measure of interrelating between the hearer and his/her predominant voice. Relating is measured across *four* scales: two concerning the hearer's perception of the relating of the voice in the form of dominance (7 Items) and intrusiveness (5 Items); and two concerning the relating of the hearer in the form of distance (7 Items) and dependence (9 Items). Each item is measured on a four-point Likert scale (0–3). The VAY has good internal consistency (α > 0.75 for all scales) and test-retest reliability (*r* > 0.7 for all scales; Hayward et al., 2008).

### Planned data analysis

All analyses were carried out using parametric analyses due to their increased power compared to non-parametric analyses and robustness to violations with relatively equal group sample sizes. When Levene's tests were significant the appropriate test statistics and alphas were reported. Prior to hypothesis testing we compared the sample characteristics of participants from each data set using chi square tests for categorical variables and ANOVAs for continuous variables. Independent-means *t-*tests were carried out in order to test each hypothesis individually, with participant gender as the grouping variable. The dependent variables of interest for each hypothesis and their respective measures were as follows: *Total Engagement, Emotional Engagement and Behavioral Engagement (BAVQ-R, Hypothesis 1); Total Resistance, Emotional Resistance, and Behavioral Resistance (BAVQ-R, Hypothesis 2); Voice Hearer Distance (VAY, Hypothesis 3)*. We then explored the potential confounding influence of age on these main analyses using ANCOVAs. Finally, we explored associations between gender and additional BAVQ-R and VAY subscales again using independent *t*-tests. Effect sizes were calculated using Cohen's *d* where *d* = 0.2 is considered a “small” effect size, 0.5 represents a “medium” effect size, and 0.8 a “large” effect size.

## Results

As data were drawn from previously published studies, it was assumed that all relevant data checks and cleaning had been administered by the respective authors. Analysis indicated no difference between samples in terms of sex [$\chi_{\left(1,\text{n}=147\right)}^{2}$ = 0.01, *p* = 0.934], age [*F*_(3, 139)_ = 0.34, *p* = 0.79; see Table 1 for mean scores], or the BAVQ-R and VAY dependent variables, except for the *behavioral engagement* subscale of the BAVQ-R [*F*_(78.35, 1076.84)_ = 2.72, *p* = 0.048]; however, Tukey's HSD and Scheffe's procedure *post-hoc* tests were unable to determine which groups differed, suggesting the study was underpowered to detect this effect (see Table 2 for BAVQ-R means and standard deviations and Table 3 for the VAY). Finally, no differences were detected between men and women with regards to age, years of voice hearing, diagnosis or type of medication taken (all *p*s > 0.1).

**Table 2:** **Means and standard deviations of BAVQ-R subscales grouped by publication**.

**Dependent variable**	**Hayward, 2003**	**Hayward et al., 2008**	**Sorrell et al., 2010**	**Dannahy et al., 2011**
Total resistance	18.41 (7.42)	20.52 (6.28)	18.23 (8.09)	21.33 (5.38)
Behavioral resistance	10.04 (4.26)	10.44 (4.16)	9.26 (5.04)	11.17 (3.90)
Emotional resistance	8.37 (3.91)	10.07 (3.37)	9.03 (3.93)	10.17 (2.35)
total engagement	5.96 (7.68)	3.7 (5.38)	5.97 (7.04)	5.96 (3.32)
Behavioral engagement	3.85 (4.07)	2.07 (2.69)	3.41 (3.32)	1.93 (2.00)
Emotional engagement	3.00 (4.49)	1.63 (2.91)	2.66 (4.51)	1.2 (2.09)
Omnipotence	11.78 (3.41)	12.52 (4.39)	11.03 (4.95)	13.57 (2.97)
Malevolence	10.96 (6.03)	11.59 (5.17)	11.22 (6.70)	12.60 (3.52)
Benevolence	5.04 (6.57)	3.70 (5.38)	4.34 (5.45)	2.03 (3.34)

**Table 3:** **Means and standard deviations of VAY subscales grouped by publication**.

**Dependent variable**	**Hayward, 2003**	**Hayward et al., 2008**	**Sorrell et al., 2010**	**Dannahy et al., 2011**
Voice dominance	–	16.41 (6.42)	14.78 (6.85)	17.13 (4.74)
Voice intrusiveness	–	10.15 (4.82)	9.03 (4.56)	10.35 (3.87)
Hearer dependence	–	6.22 (5.97)	8.34 (6.78)	8.77 (5.18)
Hearer distance	–	16.33 (4.84)	13.48 (5.52)	14.13 (4.03)

### Hypothesis testing

Independent-means *t-*tests were conducted to test each hypothesis (see Table 3 for gender mean scores and standard deviations). Due to the exploratory nature of the study, a medium effect size (0.5) was deemed to be a suitable target when carrying out a power analysis. This analysis indicated the need for 102 participants (51 per condition) in order to attain 80% power, assuming an alpha of 0.05. A total sample of 620 (310 per condition) would be required to detect small effect sizes (0.2). Therefore, the study was adequately powered to detect medium and large effect sizes but not smaller effects.

*Hypothesis 1—Female hearers will engage more with their predominant voice, when compared to male hearers—evidenced by significantly higher scores on the engagement scales of the Beliefs About Voices Questionnaire (BAVQ-R)*.

There were no gender differences in voice engagement, as measured by the BAVQ-R [*t*_(114)_ = 1.23, *p* = 0.221]. This finding would suggest that, contrary to the hypothesis, male and female voice hearers do not seem to engage differently with their voices. See Table 4 for mean responding scores grouped by gender.

**Table 4:** **Mean, standard deviations, and effect sizes for scores on the BAVQ-R and VAY grouped by gender**.

**Scale item**	***N***	**Male**	**Female**	**Sig**.	***d***
Emotional resistance	116	8.58 (4.01)	10.30 (2.56)	0.007^**^	0.52
Behavioral resistance	115	9.18 (4.76)	11.35 (3.62)	0.007^**^	0.52
Total resistance	115	17.77 (7.67)	21.64 (5.40)	0.002^**^	0.59
Emotional engagement	116	2.67 (3.92)	1.54 (3.08)	0.086	0.32
Behavioral engagement	116	3.17 (3.33)	2.45 (2.98)	0.223	0.23
Total engagement	116	5.38 (6.49)	3.98 (5.71)	0.221	0.23
Voice hearer distance	119	13.46 (4.90)	15.42 (4.40)	0.023^*^	0.42

* p < 0.05;

** *p < 0.01*.

*Hypothesis 2—Male hearers will respond to their predominant voice with greater resistance, when compared to female hearers—evidenced by significantly higher scores on the resistant scales of the Beliefs About Voices Questionnaire (BAVQ-R)*.

There was a significant difference in the extent to which participants resisted their voices. However, contrary to the hypothesis, the greatest resistance was demonstrated by female hearers [*t*_(106.18)_ = −3.15, *p* = 0.002]. Further analysis suggested that this difference was evident for both the behavioral [*t*_(109.36)_ = −2.75, *p* = 0.007] and emotional [*t*_(101.21)_ = −2.77, *p* = 0.007] aspects of resistance.

*Hypothesis 3—Male hearers will relate to their dominant voice from a position of greater distance, when compared to female hearers—evidenced by significantly higher scores on the distance scale of the Voice and You (VAY)*.

There was a significant difference in the extent to which male and female hearers related distantly to their voices. However, contrary to the hypothesis, women related from a position of significantly greater distance [*t*_(117)_ = −2.30, *p* = 0.023].

### Influence of age

Due to the possible confounding influence of age on the analyses, associations were explored between age and responses to voices. An ANCOVA test with age entered as a covariate found this variable to have little effect on the above mentioned main effects. The relative significance (*p*) values with the effects of age removed were as follows: *Emotional Resistance (BAVQ-R)* = 0.005, *Behavioral Resistance (BAVQ-R)* = 0.017, *Total Resistance (BAVQ-R)* = 0.004, *Emotional Engagement (BAVQ-R)* = 0.091, *Behavioral Engagement (BAVQ-R)* = 0.238, *Total Engagement (BAVQ-R)* = 0.206, *Voice Hearer Distance* = 0.034.

### Additional analysis

Exploratory analysis of the remaining subscales of the BAVQ-R and VAY was carried out in order to identify gender-related differences not predicted by the hypotheses. As can be seen in Table 5, women were found to appraise their voice experience as being more omnipotent [*t*_(114)_ = −2.93, *p* = 0.004], more malevolent [*t*_(110.42)_ = −2.61, *p* = 0.010], and more dominant [*t*_(118)_ = −3.03, *p* = 0.003] than men. Further, the tendency for men to appraise their voice experience as more benevolent than women approached significance [*t*_(111.89)_ = 1.96, *p* = 0.051].

**Table 5:** **Means, standard deviations, and effect sizes of additional subscales of the BAVQ-R and VAY grouped by gender**.

**Scale Item**	***N***	**Male**	**Female**	***t***	**Sig**.	***d***
Omnipotence (BAVQ-R)	116	11.17 (4.32)	13.32 (3.54)	−2.93	0.004^**^	0.55
Malevolence (BAVQ-R)	116	10.37 (5.93)	12.93 (4.61)	−2.61	0.01^*^	0.49
Benevolence (BAVQ-R)	116	4.47 (5.61)	2.70 (4.56)	1.98	0.051	0.35
Voice Dominance (VAY)	120	14.68 (6.33)	17.79 (4.87)	−3.03	0.003^**^	0.56
Voice Intrusiveness (VAY)	120	9.23 (4.54)	10.54 (3.95)	−1.69	0.093	0.31
Hearer Dependence (VAY)	120	7.60 (5.81)	8.57 (5.94)	−0.91	0.366	0.17

* p < 0.05;

** *p < 0.01*.

## Discussion

Voice hearing experiences are being considered within relational frameworks, and empirical studies suggest that “relationships” with voices share many similarities with social relationships. Given that differences exist in the ways that males and females relate socially (females seeking greater intimacy and closeness; males seeking greater distance), this study explored the possible influence of gender upon hearer responses within relationships with voices. It was hypothesized that female hearers would seek to engage with their voices and exhibit strong emotional reactions to them, whilst male hearers would seek to resist their voices and keep them at a distance. Data were drawn from four published studies involving clinical voice hearers and, contrary to expectations, female hearers did not engage with their voices more than male hearers. Instead, female hearers responded to their voices with significantly greater resistance and sought greater distance from them.

The findings from this study would therefore appear to contradict the gender differences reported in the literature on social relating. Rather than exhibiting a stronger emotional reaction and seeking to express greater intimacy with voices, female hearers exhibited greater resistance to their voices and attempted to withdraw from them. However, a review of the items within the “emotional resistance” scale of the BAVQ-R suggests that an alternative interpretation might be available. The scale contains items that require the respondent to rate how much voices make them feel frightened, down, angry, and anxious. Responding affirmatively to these items could be interpreted as emotional reactivity, rather than resistance. The resistance that is captured by the “behavioral resistance” scale (e.g., I try to stop my voice/I tell my voice to leave me alone) could then be considered as an attempt to push voices away in response to this strong emotional reaction, as the hearer seeks to distance themselves from the source of these strong and unpleasant emotions.

This interpretation of resistant behavior being triggered by an emotional reaction in female hearers is consistent with what is known about the negative and personally salient content of voice utterances. The content of voices often relates to past abuse and traumatic events (Hardy et al., 2005) and females who have been diagnosed with psychosis (e.g., Fisher et al., 2009; Shah et al., 2014) and hear voices (Daalman et al., 2012) more frequently report experiences of childhood abuse. If, by virtue of a hearer's gender, they are disposed toward a stronger emotional reaction (e.g., Searle and Meara, 1999), an appropriate behavioral response would be to try and eliminate the stimulus (voice utterances) that is generating the distressing emotion. If the stimulus cannot be eliminated, an alternative strategy might be to try and withdraw from the stimulus and create some distance between oneself and the voice. Both of these approaches have been utilized instinctively (Tsai and Ku, 2005) and therapeutically (Tarrier et al., 1990) with people who hear distressing voices with some effect, but may not generate positive outcomes in the longer term (Tarrier et al., 1993). Indeed, such “fight” and “flight” strategies have been associated with a range of negative outcomes (see Shawyer et al., 2013 for a review).

Conceptually, the interpretation of this study's findings both supports the central role of emotion in (at least) maintaining psychotic experiences (Garety et al., 2001), and also elaborates the cognitive model of voices. Appraisals of/beliefs about voices are the central tenet of the model, whereby the hearer's appraisal of their voice (as all powerful and having malevolent intent) has a significant influence upon their emotional and behavioral responses. The findings from this study suggest that the behavioral (resistant) response of the (female) hearer may (in part) be triggered, not wholly by the appraisal of the voice, but also by their emotional response—thereby elaborating the “consequences” (C) part of the ABC model by making explicit the association between emotional and behavioral responses. The findings may also have implications for the role of appraisal within the model, as emotional responses to voices could be stimulated by negative and personally salient content of voices, without appraisals exerting a mediating influence. This possibility is consistent with the findings of Close and Garety (1998) that voice content (As) and hearer responses (Cs) were consistent for all of the participants within their study. This direct relationship between As and Cs may also contribute to an understanding of the limited impact of CBT upon voice distress, despite successfully modifying beliefs about voices (e.g., Birchwood et al., 2014).

A further interpretation of this study's findings is offered by the additional exploratory analysis. Female hearers perceived their voices as significantly more powerful (omnipotent and dominant) and malevolent, compared to the perceptions of males. This might suggest that the emotional response of female hearers is influenced by their cognitive appraisals of the voices, corroborating the central tenet of the cognitive model (Chadwick and Birchwood, 1994). From an interpersonal perspective, the distribution of power across genders may be relevant here. Where power imbalances are evident in romantic relationships, males are perceived to be more powerful (Felmlee, 1994; Simpson et al., 2015). This imbalance may interact with the most common perception of voice gender (male—McCarthy-Jones et al., 2014a) and create a sense of disempowerment for female hearers which provokes a strong emotional reaction and resistant response.

Given the ability of the content and appraisals of voices to trigger an emotional response in hearers, and the possibility that female hearers might react more strongly to these emotions, what does this say about male hearers? Is it the case that voices trigger an emotional response of less intensity in males, or do they react less strongly to an emotional response of a similar intensity? Known gender differences in adult attachment might suggest that men, who are more likely to have avoidant attachment styles, might experience less negative affect in responses to voices. However, models of adult attachment also suggest that in the context of extreme stress, avoidant attachment strategies can “break down” and are no longer effective, resulting in overwhelming negative affect (Mikulincer and Shaver, 2010).

### Clinical implications

The clinical implications of the findings from this study seem both broad and narrow. From a broad perspective, there is a suggestion that closer attention should be paid to the links between emotional and behavioral responses to voices, and their association with the negative and personal salience of the content of voice utterances. Making these connections within an individual formulation will normalize and validate the responses of the hearer (Corstens and Longden, 2013), and identify targets for intervention. These targets may include the behavioral responses of the hearer—and the possible evidence that different responses can generate to support the development of new (and less distressing) meaning.

If we know that passive/submissive and aggressive responses to voices, whilst understandable and instinctive, can maintain distress (Shawyer et al., 2013), what alternatives might exist? One option might be to do nothing—to merely notice the voice and locate one's attention elsewhere. Such a response is practiced and developed within mindfulness-based approaches, and can generate new meaning to support beliefs about the self as having some control, even when voices are around. Hallucination-proneness has been reported to be negatively correlated with mindfulness (Perona-Garcelán et al., 2014) and preliminary evidence has been generated for the potential of mindfulness-based approaches with voice hearers (Dannahy et al., 2011; Strauss et al., 2015). A further option might be to respond assertively—talking back to the voice in a manner that respects both the self and the voice; letting the voice know how it's making one feel, and offering evidence to support a more balanced (and less negative) view of self. A novel therapy is being developed by Hayward et al. (2009) to explore this way of responding and a pilot RCT is underway (Hayward et al., 2014). A final option for responding differently might involve responding in an “anti-complementary” way (Thomas et al., 2009), e.g., responding in a friendly manner to a derogatory voice.

A narrower implication of this study's findings concerns the fit of interventions to the gender of the hearer. Given their stronger emotional reaction to voices, and the greater extent to which they might be drawn into responding to the voice in a resistant manner, how might the alternative ways of responding suggested above be more or less effective for female hearers? Mindfulness-based interventions seem an obvious candidate as the invitation within this approach is not to react to voices. If a female hearer feels as if she is helplessly drawn into responding passively to her voice, what meaning might she give to her ability to bring her attention away from voices, and not get caught-up in listening to them, despite their insistence that she do so? What impact might this response have upon her sense of self? Future research might benefit from exploring the experience of hearers within mindfulness-based interventions, with a focus upon the effectiveness of the approach across genders.

## Limitations

There are a several limitations to this study and analyses which pinpoint areas for further research. First, although this study benefits from a pooled data set, the total sample size is relatively small and possibly not powered to detect small between-group effects. Second, the multiple testing will have increased the likelihood of Type I errors. However, due to the exploratory nature of our study, we did not want to be too conservative and apply bonferroni corrections (Perneger, 1998). Third, the voice hearers who participated in the studies may not be representative of all voice hearers, for example, participants may be more willing or open to describing their voice hearing experiences than other voices hearers or by virtue of the studies' inclusion criteria participants are unlikely to have been in more acute phases of distress. Relatedly, there is growing recognition that there are different subgroups of voice hearers (McCarthy-Jones et al., 2014b) and gender and responses to voices may be more relevant in some groups compared to others. Fourth, although we explored and rejected the potentially confounding influence of age on our analyses, there may be other important but unmeasured confounds which better explain the association between gender and response to voices, including severity of voices and voice-related distress. Finally, our study did not investigate mechanisms which might account for associations between gender and ways of relating to voices. Attachment theory is a key interpersonal theory and may be an important variable to investigate in developing understanding of how males and females relate to their voices as well as other key people in their lives (Berry et al., 2012).

To overcome these limitations, we recommend that future studies should include large representative samples which enable subgroup analyses of different types of voice-hearing experiences. These studies should include multi-dimensional measures of voices which assess voice-related severity and distress as well as other important factors, including beliefs about, and ways of relating to, voices and adult attachment style.

## Author contributions

All authors listed, have made substantial, direct and intellectual contribution to the work, and approved it for publication.

### Conflict of interest statement

The authors declare that the research was conducted in the absence of any commercial or financial relationships that could be construed as a potential conflict of interest.
